# Multiple reversible encephalitic attacks: a rare manifestation of neuronal intranuclear inclusion disease

**DOI:** 10.1186/s12883-020-01712-5

**Published:** 2020-04-08

**Authors:** Mingming Li, Kai Li, Xin Li, Yun Tian, Lu Shen, Guode Wu, Zaiqiang Zhang, Weian Chen

**Affiliations:** 1 Department of Neurology, Lanzhou University Second Hospital, Lanzhou, 730000 Gansu China; 2 Department of Neurology, Beijing Hospital, National Center of Gerontology, Beijing, 100730 China; 3 Department of Neurology, Xiangya Hospital, Central South University, Changsha, 410008 Hunan China; 4 Department of Neurology, Beijing Tiantan Hospital, Capital Medical University, Beijing, 100050 China; 5 Department of Neurology, First Affiliated Hospital of Wenzhou Medical University, Wenzhou, 325000 Zhejiang China

**Keywords:** Neuronal intranuclear inclusion disease, Multiple reversible encephalitic attacks, Intranuclear inclusion, Skin biopsy, GGC repeat expansions

## Abstract

**Background:**

Neuronal intranuclear inclusion disease (NIID) is a rare neurodegenerative condition characterized by the loss of neurons and the presence of eosinophilic nuclear inclusions in the central and peripheral nervous system, skin and visceral organs. In this paper, we present a case of NIID with recurrent encephalitic attacks that remained stable and nonprogressive for seven years; no such case has previously been reported.

**Case presentation:**

A 63-year-old female was hospitalized due to light-headedness, vomiting, unstable gait and cognitive impairment. Seven years prior, she had experienced an episode of light-headedness, central facial paralysis, unstable gait, aphasia, nausea, vomiting and loss of consciousness. She regained consciousness within 12 h, and her other symptoms were completely resolved within one week. During the present hospitalization, a brain magnetic resonance imaging (MRI) examination detected high signal intensity on diffusion-weighted imaging (DWI) of the bilateral frontal grey matter–white matter junction. We reviewed the patient’s previous MRI results and found that she had also had high signal intensity on DWI of the bilateral frontal grey matter–white matter junction seven years prior. In the intervening seven years, the high signal intensity in the frontal lobes had spread along the grey matter–white matter junction, but the deep white matter remained unaffected. Skin biopsy was performed, and intranuclear inclusions were found in adipocytes, fibroblasts and sweat gland cells. GGC repeat expansions in the *NOTCH2NLC* (Notch 2 N-terminal like C) gene confirmed the diagnosis of NIID. She received supportive treatment such as nutrition support therapy and vitamin B and C supplementation, as well as symptomatic treatment during hospitalization. The patient’s symptoms were completely relieved within one week.

**Conclusion:**

This is a detailed report of a case of NIID with multiple reversible encephalitic attacks, diagnosed by clinical symptoms, intranuclear inclusions, characteristic DWI signals, and genetic tests.

## Background

NIID is a rare, slowly progressing neurodegenerative disease characterized by eosinophilic intranuclear hyaline inclusions in cells of the central and peripheral nervous system (including the autonomic nervous system) and the visceral organs [1]. GGC repeat expansion in the 5′ untranslated region (5′ UTR) of the *NOTCH2NLC* (Notch 2 N-terminal like C) gene is associated with the mechanism of NIID [2]. NIID may occur from infancy to old age. According to the age of onset, NIID can be categorized as infantile, juvenile, or adult. Sporadic NIID emerges between 51 and 76 years of age [3]. Dementia (94.7%), muscle weakness (27%), sensory disturbances (28.6%), autonomic nervous dysfunction, ataxia, epilepsy, paroxysmal disturbance of consciousness (39.5%) and parkinsonism are frequently observed in adult-onset NIID patients [4]. While adult-onset NIID is characterized by slowly progressing dementia, it may also present with acute symptoms including stroke-like episodes and epileptic seizures [5]. Dementia and limb weakness are the initial and main clinical manifestations of adult-onset NIID. In most NIID patients over 40 years old, dementia is the first symptom [6]. Diagnosis of the disease formerly required an autopsy but can now be established by skin biopsy and genetic examination [7].

We report a case of NIID with multiple reversible encephalitic attacks, in which the patient experienced two encephalitic episodes followed by complete remission of symptoms. As illustrated by this case, clinicians need to be alert to the possibility of NIID when patients have reversible encephalitic attacks.

## Case presentation

**Fig. 1:**
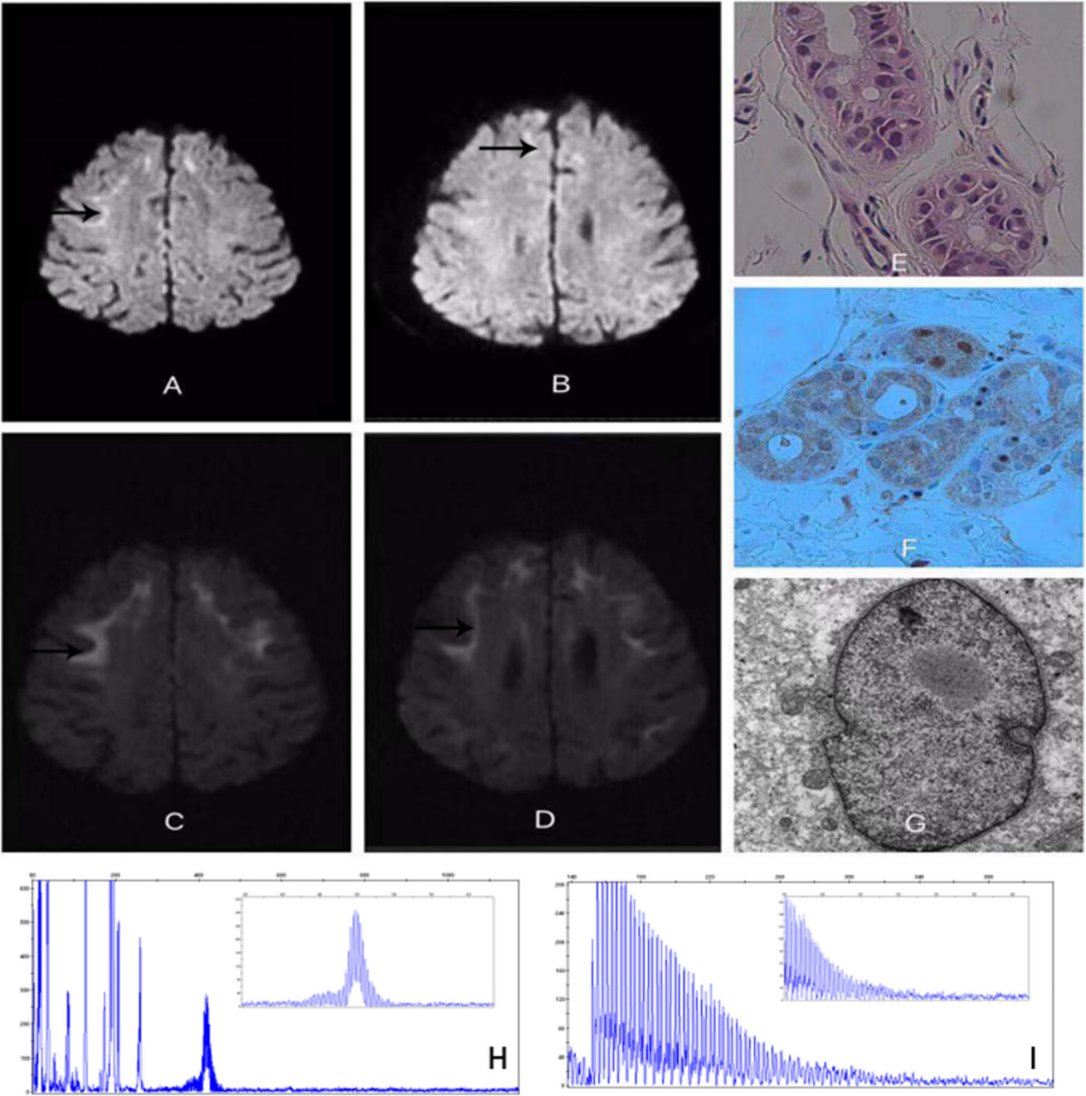
Brain magnetic resonance imaging demonstrated high-intensity areas in the cerebral white matter on diffusion-weighted imaging seven years before the most recent hospitalization (**a**, **b**). Brain magnetic resonance imaging revealed high-intensity areas in the cerebral white matter and the grey matter–white matter junction on diffusion-weighted imaging (**c**, **d**). Skin biopsy samples were subjected to immunohistochemical staining. There were some circular or approximately circular structures deeply stained with 4-dimethylaminoazobenzene in the membranes of sweat gland cells (**e**: haematoxylin–eosin staining, × 200; **f**: anti-p62 immunohistochemical staining, × 200; **g**: electron micrograph). This patient had 96 GGC repeats in *NOTCH2NLC* (electropherograms of the GC-rich PCR assay [**h**] and the repeat-primed PCR assay [**i**])

A 63-year-old woman was admitted to hospital with an acute neurological episode. The patient’s complaints were light-headedness, vomiting, unstable gait and cognitive impairment. She had been healthy except for a similar attack seven years prior and had no family history of similar symptoms. Upon admission, the patient’s body temperature was 36.7 °C, and her blood pressure was 122/86 mmHg. Neurological examination revealed no abnormalities in cranial nerve function or muscle strength. The bilateral knee reflex and Achilles tendon reflex were diminished. The biceps and triceps reflexes were normal. The bilateral plantar responses were flexor. Her gait was wide-based and ataxic. She did not have increased muscle tone or bradykinesia. Her complete blood count was normal, as were her liver and kidney function. Neuropsychological examinations revealed mild cognitive impairment: 24/30 on the Mini-Mental State Examination (MMSE), 25/30 on the Montreal Cognitive Assessment (MoCA), 10 on the Hamilton Depression Scale (HAMD), 24 on the Hamilton Anxiety Scale (HAMA), 3 on the Neuropsychiatric Inventory (NPI) and 33 on the Scales for Outcomes in Parkinson’s Disease–Autonomic (SCOPA-AUT). The decreases in the MMSE and MoCA scores were caused mainly by reductions in the patient’s short-term memory and computational ability. Brain MRI revealed high signal intensity in the bilateral frontal grey matter–white matter junction on DWI (Fig. 1 c, d). Cerebrospinal fluid examination revealed mildly elevated levels of protein (0.55 g/l) and glucose (4.5 mmol/l), an absence of red blood cells (0 × 10^9^/l), and a normal leukocyte count (3 × 10^6^ monocytes/l). The patient’s nerve conduction studies were normal. An electroencephalogram (EEG) showed no epileptic discharge. A review of the patient’s brain MRI results from 2011 revealed the presence of a high signal intensity on DWI of the bilateral frontal grey matter–white matter junction (Fig. 1 a, b). According to a detailed inquiry into the patient’s medical history when she was 56 years old, she had experienced light-headedness, right lower quadrant facial droop, unstable gait, aphasia, nausea, vomiting and loss of consciousness. She was admitted to another hospital, where she regained consciousness within 12 h of symptom onset. She received symptomatic treatment, and all other symptoms were completely resolved within one week. The MRI scan showed disease progression compared to the imaging from seven years earlier. Skin biopsy revealed intranuclear inclusions in adipocytes, fibroblasts and sweat gland cells (Fig. 1 e, f, g), which was compatible with NIID. Genetic examination using GC-rich PCR (GC-PCR) and repeat-primed PCR (RP-PCR) found that the patient had 96 GGC repeats in the 5′ UTR of *NOTCH2NLC* (Fig. 1 h, i). She was not a carrier of the *fragile X mental retardation 1* (*FMR1)* premutation.

During hospitalization, the patient was given symptomatic treatment such as anti-dizziness and anti-vomiting treatment as well as supportive treatment such as nutrition support therapy and vitamin B and C supplements during hospitalization, and her symptoms were completely relieved within one week.

In this case study, the patient had two acute encephalitic attacks that were manifested mainly as loss of consciousness, neuropsychological impairments, gait disorders, autonomic dysfunction, etc. Normal body temperature, complete blood count, and cerebrospinal fluid cell counts helped exclude infectious encephalitis. The characteristic high signal intensity on DWI alerted the physicians to perform skin biopsy and genetic testing. The skin biopsy revealed intranuclear inclusions in adipocytes, fibroblasts and sweat gland cells; this set of features was compatible with NIID. A negative test result for the *FMR1* gene premutation excluded fragile X–associated tremor ataxia syndrome (FXTAS). The expansion of repeats in the 5′ UTR of the *NOTCH2NLC* gene confirmed the diagnosis of NIID. At the time of this report, the patient’s outcome is good.

## Discussion and conclusions

This case study describes a patient with NIID who presented with two acute reversible encephalitic episodes over seven years. The patient was diagnosed on the basis of characteristic DWI signals, intranuclear inclusions, a negative *FMR1* gene premutation and expansion of repeats in the *NOTCH2NLC* gene.

In light of the patient’s GGC repeat expansion within human-specific *NOTCH2NLC* and the absence of *FMR1* gene premutation, she was diagnosed with sporadic adult-onset NIID [2]. FXTAS is a late-onset neurodegenerative disorder that is similar to NIID in terms of clinical and neuropathological features [8]. We excluded the diagnosis of FXTAS through *FMR1* genetic testing, which showed that the number of CGG repeats was within the normal range (< 44 repeats).

Over the seven-year period of disease progression, high DWI signal spread along the grey matter–white matter junction, but the deep white matter was not affected. The signal abnormality progressed continuously once it appeared, which is consistent with previous reports [9].

Due to the highly variable nature of the clinical symptoms, NIID is considered a heterogeneous disease [10]. Most of the previously reported cases showed a degenerative disease course or presented as encephalitis that subsequently deteriorated. One previous case reported a reduced level of consciousness and recurrent vomiting, though the patient’s symptoms continued to progress, which differs from our case [11]. Although multiple reversible encephalitic attacks have been described in case reports previously, some symptoms such as dysarthria recovered completely afterwards, but there were some residual symptoms such as impaired memory and word recall, saccadic pursuit eye movements and mild weakness of the left lower extremity [12–14]. In this case, we report a patient with NIID who presented with recurrent loss of consciousness and cognitive impairment that completely recovered within a short time. An important finding was the lack of residual symptoms from the paroxysmal encephalitic episodes. Clinicians should be aware of these findings, and the possibility of NIID should be considered when these symptoms are present. To the best of our knowledge, NIID with multiple reversible attacks has not previously been reported.

NIID is characterized pathologically by the presence of eosinophilic intranuclear inclusions in neuronal and glial cells [3, 15]. Factors contributing to the development of NIID can include abnormal protein accumulation and dysfunction of the intranuclear protein degradation system. The nuclear inclusions (NIs) in the biopsy specimens are immunopositive for ubiquitin and p62 [16]. p62 is an adaptor molecule for the selective autophagic degradation of ubiquitinated proteins [17]. According to recent research, p62 can shuttle into the nucleoplasm and plays an important role in the degradation of intranuclear proteins [18]. It remains unclear what role p62 may play in the pathogenesis of NIID. The inclusions do not seem to cause cell death, but they can disrupt neuronal function [19]. In a study by Nakano [20], ubiquitin- and p62-positive intranuclear aggregates appeared in both controls and NIID cases. However, activated nuclear bodies (NBs) have never been observed in NIID cases, which suggests that the p62- and PML-positive structures might differ in ultrastructural terms between normal and NIID cases. The aberrant response of NBs to stress might lead to fluctuations in clinical symptoms [21]. Accordingly, the dysfunction of NBs or abnormal proteins could be related to the pathogenesis of NIID.

Recently, several studies suggested that NIID was associated with *NOTCH2NLC* gene alterations [7, 22]. *NOTCH2NLC* is highly expressed in various populations of radial glia and is associated with evolutionary expansion of the human cerebral cortex [23]. *NOTCH2NLC* lacks the N-terminal signal peptide and has a 2-bp deletion just downstream of the *NOTCH2* start codon. Moreover, by long-read sequencing, GGC repeat expansion in the 5′ region of *NOTCH2NLC* was identified in all patients with familial NIID as well as those with sporadic NIID. Sone et al. found that the change in the number of repeats ranged from 6 to 61 in control subjects and 71 to 183 in NIID patients [22]. In a study by Tian and colleagues, the number of GGC repeat expansions was less than 40 in healthy subjects and 66 to 517 in NIID patients. Additionally, GGC repeat expansions have been identified as the genetic cause of NIID pathogenesis in the Chinese population [2]. Abnormal GGC duplication in the 5′ UTR region of the human-specific *NOTCH2NLC* gene is the main cause of NIID and related diseases [7]. GGC repeat expansions may cause neuronal toxicity that leads to NIID [22].

In summary, we present a case of NIID with multiple reversible encephalitic attacks; such a case has never been previously reported. The case was diagnosed by intranuclear inclusions on skin biopsy and by genetic testing; these diagnostics were carried out in response to high-intensity DWI signal at the grey matter–white matter junction. Clinicians need to be alert to the possibility of NIID when patients exhibit reversible encephalitic attacks and intense DWI signal at the grey matter–white matter junction.

## Data Availability

The datasets used and/or analysed during the current study are available from the corresponding author on reasonable request.
